# The Warburg Effect Occurs Rapidly in Stimulated Human Adult but Not Umbilical Cord Blood Derived Macrophages

**DOI:** 10.3389/fimmu.2021.657261

**Published:** 2021-04-13

**Authors:** Cilian Ó Maoldomhnaigh, Donal J. Cox, James J. Phelan, Fergal D. Malone, Joseph Keane, Sharee A. Basdeo

**Affiliations:** ^1^ TB Immunology Group, Department of Clinical Medicine, Trinity Translational Medicine Institute, St James’s Hospital, Trinity College Dublin, The University of Dublin, Dublin, Ireland; ^2^ Obstetrics & Gynecology, Royal College of Surgeons in Ireland, Rotunda Hospital, Dublin, Ireland

**Keywords:** immunometabolism, macrophage, glycolysis, human, neonatal

## Abstract

The Warburg effect, defined as increased glycolysis and decreased oxidative phosphorylation, occurs in murine macrophages following LPS stimulation and is required for activation. There are differences between human and murine macrophage metabolic responses to stimulation, with peak metabolite concentrations occurring earlier in humans than mice. Complex changes occur in the human immune system with age, resulting in the very young and the very old being more susceptible to infections. Anti-bacterial immune responses in umbilical cord immune cells are considered deficient but there is a paucity of data on the role that metabolism plays. We hypothesized that metabolic responses in human macrophages occur early during activation. In addition, we hypothesized that umbilical cord derived macrophages have an altered immunometabolic response compared with adult macrophages. We demonstrate that adult and cord blood monocyte derived macrophages (MDM) immediately increase glycolysis in response to stimulation with LPS or *Mycobacterium tuberculosis* (Mtb), however only adult MDM decrease oxidative phosphorylation. At 24 hours post stimulation, glycolysis remains elevated in both adult and cord blood MDM, oxidative phosphorylation remains unchanged in the cord blood MDM and has normalized in the adult MDM stimulated with Mtb. However, LPS stimulated adult MDM have increased oxidative phosphorylation at 24 hours, illustrating differences in metabolic responses to different stimuli, time-dependent variation in responses and differences in macrophage metabolism in adults compared with umbilical cord blood. We compared the phenotype and function of macrophages derived from adult or cord blood. Cord blood MDM secreted less TNF following Mtb stimulation and more IL-6 following LPS stimulation compared with adult MDM. Our findings demonstrate that whilst cord blood MDM exhibit an immediate increase in glycolytic flux in response to stimulation, similar to adult MDM, cord blood MDM do not concomitantly decrease oxygen consumption. This indicates that adult macrophages shift to Warburg metabolism immediately after stimulation, but cord blood macrophages do not. Understanding the differences in the metabolic profiles of macrophages over a human lifetime will enable the translation of immunometabolism into effective immuno-supportive therapies that could potentially be targeted at vulnerable populations, such as the very old and the very young.

## Introduction

Murine macrophages have been shown to increase glycolysis and decrease oxidative phosphorylation following lipopolysaccharide (LPS) stimulation (1, 2). This altered metabolism in activated murine macrophages is akin to the altered metabolic function first observed in tumor cells by Otto Warburg (3) and is thus, termed the ‘Warburg effect’. More recent findings linking changes in the metabolic function of immune cells to their ability to mount an effective immune response (4–6), has led to the development of a field of research known as immunometabolism. The majority of work published to date in macrophages has been carried out in murine bone marrow derived macrophages (BMDM), which has been crucial to the detailed mechanistic understanding of these biochemical processes. However, there is a paucity of immunometabolic data generated in primary human macrophages (7). Furthermore, there are seemingly contradictory findings between human and murine macrophage research, with more research required to fully establish the metabolic features of activated human immune cells (7, 8).

A study directly comparing murine BMDM and human monocyte derived macrophages (MDM) replicated the established increase in murine extracellular acidification rate (ECAR), a correlate of glycolysis, and decrease in oxygen consumption rate (OCR), correlated to mitochondrial oxidative phosphorylation, in response to LPS (8). In contrast, human MDM had reduced ECAR and no change in OCR 16 hours after LPS stimulation (8). It is difficult to draw accurate conclusions from these findings that illustrate stark differences in human and mouse immunometabolism; however, these differences are not unexpected given the striking disparities between human and murine susceptibility to LPS toxicity (9). Other studies examining itaconate, an immunomodulatory derivative of a tricarboxylic acid cycle intermediate, which increases after macrophage activation, found that the peak in human MDM occurred earlier than in murine BMDM (10, 11). Thus, we postulated that the kinetics of metabolic flux may occur early in human macrophages responding to stimulation.

These disparities may hamper efforts to translate this knowledge into effective therapeutic drugs. Adding further to this complexity, is the changing function of human immune responses over a lifetime. The role of metabolism in immune defects seen at the extremes of age is an area of ongoing investigation (12–14). Newborn babies are more susceptible than adults to a variety of bacterial infections, including from gram negative organisms (containing LPS) and from intracellular pathogens such as *Mycobacterium tuberculosis* (Mtb) (15, 16), the causative agent of Tuberculosis (TB). Our laboratory has previously shown that adult human macrophages shift their metabolic function toward aerobic glycolysis after Mtb infection (17–19), a finding replicated in other centres (20, 21). Furthermore, research examining monocytes and MDM from umbilical cord blood have shown them to be functionally impaired in relation to adult comparators, although the data can be contradictory (22–24). There is a lack of published data on metabolic activation in cord blood MDM, with only one study showing diminished glycolysis in polarized MDM (25).

In order to exploit the therapeutic potential of manipulating metabolic pathways, basic human cellular research is required to build on the growing literature generated from murine studies in immunometabolism. We hypothesized that the kinetics of human macrophage immunometabolic responses would differ from the well-established murine pattern and that the altered immune phenotype seen in newborns compared with adults may be a result of underlying differences in metabolism. Our work demonstrates, for the first time, that adult MDM undergo a rapid shift to Warburg metabolism upon stimulation; with an increase in glycolysis and a decrease in oxidative phosphorylation, which peaks about 2.5 hours following stimulation and resolves 5 hours post stimulation. Cord blood MDM also rapidly increase glycolysis but do not decrease oxygen consumption in response to stimulation with either LPS or Mtb. Glycolysis remains increased in both adult and cord blood MDM 24 hours post stimulation. Cord blood MDM have similar expression of extracellular markers of activation and phenotype as adult MDM but produce less TNF following Mtb and more IL-6 following LPS stimulation.

These data are the first to show that the Warburg effect occurs in adult human macrophages. Furthermore, our work indicates that human macrophage metabolic flux and function in response to stimulation may change during childhood development and ageing.

## Materials and Methods

### MDM Cell Culture

Umbilical cord blood (UCB) samples were drawn from the placental umbilical cord immediately following delivery of the placenta. All babies were healthy term infants of normal birthweight who were delivered by elective Caesarean section. All mothers were well throughout pregnancy and had no co-morbidities. Informed consent was obtained from the mother prior to delivery. Ethical approval was granted by the Rotunda Ethics Committee. Adult samples were obtained with consent from the buffy coats of healthy donors (aged between 18-69) from the Irish Blood Transfusion Services. Peripheral blood mononuclear cells (PBMC) were isolated by density-gradient centrifugation over Lymphoprep (StemCell Technologies). Cells were washed, resuspended at 2.5x10^6^ PBMC/ml in RPMI (Gibco) supplemented with 10% AB human serum (Sigma-Aldrich) and plated on to non-treated tissue culture plates (Costar). Cells were maintained in humidified incubators for 7 days at 37°C and 5% CO_2_. Non-adherent cells were removed by washing every 2-3 days. MDM cultures were routinely >90% pure based on co-expression of CD14 and CD68, analyzed by flow cytometry.

### MDM Stimulation

MDM were stimulated on day 7 with LPS (Sigma-Aldrich; 100 ng/ml) or irradiated H37Rv (iH37Rv; gifted by BEI Resources). Macrophages were infected with a multiplicity of infection (MOI) of 1-10 bacteria per cell (approximately 70% infected). Briefly, cryopreserved iH37Rv was thawed, sonicated and passed through a 25-gauge needle prior to adding it to cells. Donor variation in phagocytosis of Mtb was adjusted for by Auramine O staining, as previously described (26). For the experiments where analysis was undertaken after 24 hours, the extracellular bacteria were washed off after 3 hours, pelleted by centrifugation and half of the volume of supernatant was placed back on the well. Macrophages were then incubated for a further 21 hours in humidified incubators at 37°C and 5% CO_2_. Unstimulated macrophages were assayed in parallel as controls. The same MOI defined by Auramine O staining was also undertaken for the live analysis of Mtb stimulation in the Seahorse XFe24 Analyzer with the calculated volume of Mtb added to the ports of the Seahorse XFe24 Analyzer.

### Expression of Cell Surface Markers by Flow Cytometry

MDM were placed in ice cold PBS and incubated at 4°C on ice for 30 minutes. Cells were removed from the plastic by gentle scraping, Fc blocked with Human TruStain FcX (BioLegend) and stained with zombie NIR viability dye and fluorochrome-conjugated antibodies specific for CD14 (FITC), CD68 (PE), CD83 (PerCP-Cy5.5), CD80 (PE-Cy7), CD86 (BV410), CD40 (BV510), and HLA-DR or MMR (APC; all BioLegend). Cells were fixed with 2% PFA and acquired on a BD FACS Canto II. Unstained cells and FMO controls were used to normalize for background staining and to set gates. Data were analyzed using FlowJo software.

### Metabolic Assays Using the Seahorse XFe Analyzer

Macrophage metabolic function was assessed using the Seahorse XFe Analyzer (Agilent). After 7 days of adherence purification in the presence of 10% human serum, the medium was replaced with ice-cold PBS and MDM were placed on ice at 4°C for 30 minutes. The MDM were then gently scraped and counted using trypan blue to exclude dead cells. MDM (1x10^5^ cells/well) were re-plated onto Seahorse plates, as previously described (18). The extracellular acidification rate (ECAR) and the oxygen consumption rate (OCR), surrogates for glycolysis and oxidative phosphorylation respectively, were measured 3 times every 9 minutes to establish baseline rates. For the mitochondrial stress test experiments, MDM were stimulated with Mtb or LPS 24 hours prior to analysis in the Seahorse XFe Analyzer. The Mito Stress Test was performed as per manufacturer’s instructions (Agilent); with the sequential addition of oligomycin (1 μM), FCCP (1 μM) and antimycin-A/rotenone (0.5 μM). ECAR and OCR readings were sampled every 9 minutes. For experiments where cells are stimulated and monitored in real time, the Seahorse XFe Analyzer injected Seahorse medium, Mtb or LPS into assigned wells after 30 minutes. The ECAR and OCR readings were then continually sampled every 9 minutes in real time for 8 hours. Due to the longer timeframe of this assay, every second data point was plotted (approximately every 18 minutes) and the graph was terminated at 400 minutes. Percentage change was calculated versus the 3rd baseline reading to normalize for donor and experimental variation. Differences in cell density were corrected for by crystal violet normalization, as previously described (19). Data are expressed as percentage change from the 3^rd^ baseline reading to facilitate comparisons between the metabolic profile observed in real time and the profile 24 hours post stimulation.

### Cytokine Production by ELISA

The concentrations of IL-1β, IL-10, TNF, IL-6, IL-8, IL-12p70 and IFN-γ present in the supernatants were quantified using Meso Scale Discovery (MSD, Rockville, MD), according to the manufacturer’s protocol.

### Statistical Analysis

Statistical analyses were performed using GraphPad Prism 9 software. Statistically significant differences between two normally distributed groups were determined using Student’s paired or unpaired t-tests (as appropriate) with two-tailed *P*-values. Differences between three or more groups were determined by one-way ANOVA with Tukey’s multiple comparisons tests. Differences between two or more groups containing more than one variable were determined by two-way ANOVA with Sidak’s multiple comparisons tests. *P*-values of <0.05 were considered statistically significant and denoted using an asterisk.

## Results

### Cord Blood MDM Show Similar Cell Surface Marker Expression Compared to Adult MDM Following Stimulation With Mtb or LPS

Differences in adult and cord blood MDM have not been consistently reproduced, in part because of the numerous methods used to differentiate human macrophages from monocytes (27–29). We first examined the morphology, phagocytosis and purity of the adult and cord blood macrophages after differentiation using a well-established method of adherence purification in 10% human serum (17–19, 26, 30, 31). PBMC were isolated from adult buffy coats or from umbilical cord blood which was collected immediately following delivery. MDM were adherence purified for 7 days in RPMI with 10% human serum and non-adherent cells were washed off on days 2 and 5. Similar morphology was seen under light microscopy for both adult and cord blood MDM after 7 days of differentiation (**Supplementary Figure 1A**). The multiplicity of infection of Mtb was calculated for every donor in order to correct for variances in inter-donor phagocytosis. MDM cultures from adult and cord blood were both routinely >90% pure, as demonstrated by flow cytometry (**Supplementary Figures 1B, C**). Cord blood MDM were more prone to death than adult MDM after the cold scraping method of detaching MDM from plastic (**Supplementary Figure 1D**). This was carefully adjusted for throughout our experiments, as described in the methods section.

We examined the expression of cell surface markers of activation, associated with pro-inflammatory “M1” or anti-inflammatory “M2” type functions. We performed flow cytometry 24 hours after stimulation with Mtb or LPS on adult and cord blood MDM. MDM were harvested by placing in ice-cold PBS at 4°C for 30 minutes prior to gentle scraping. Cells were Fc blocked and stained with Zombie NIR viability dye and fluorochrome-conjugated antibodies specific for CD68, CD14, CD40, CD80, HLA-DR, CD83 and CD206 (mannose receptor; MMR) prior to acquisition by flow cytometry. MDM were gated on the basis of forward and side scatter, doublets and dead cells were excluded, and macrophages were identified as CD68^+^ CD14^+^ as shown in the gating strategy (**Supplementary Figure 1B**).

**Figure 1:**
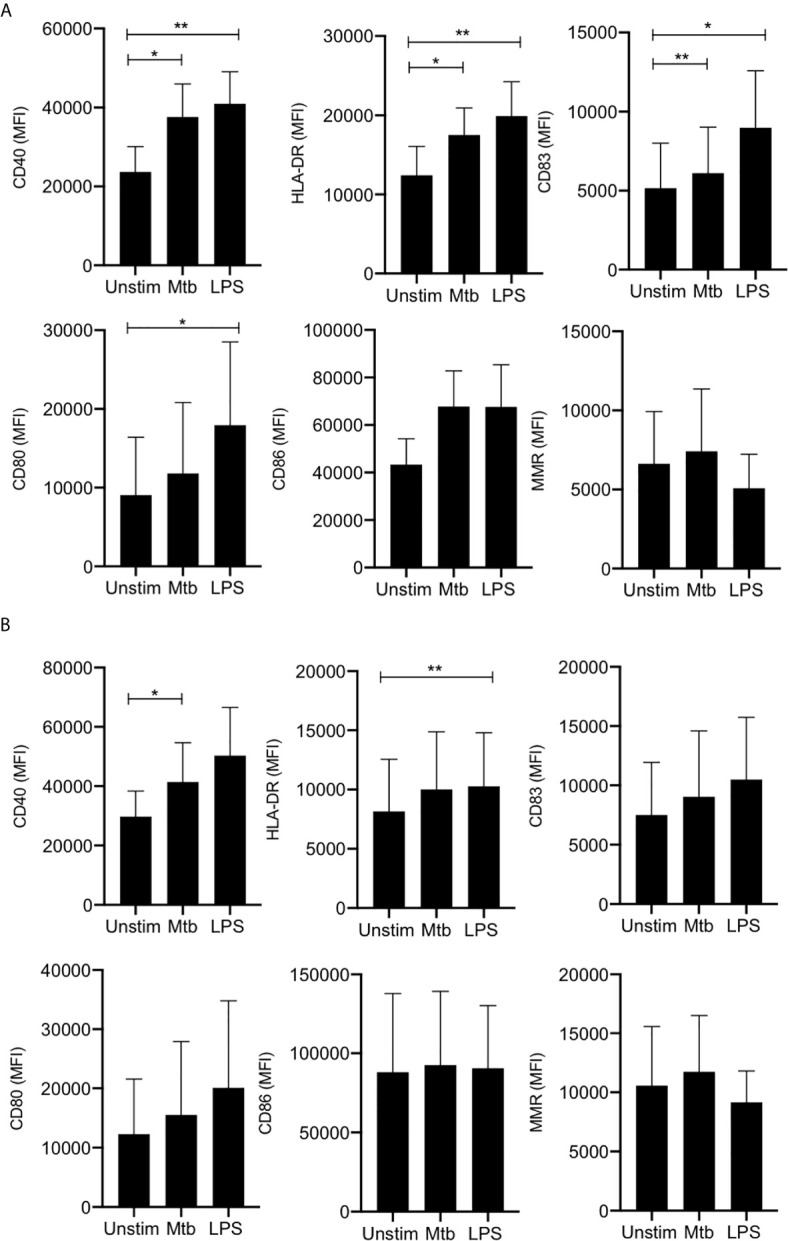
Adult and cord blood MDM have similar expression of activation and phenotypic markers. PBMC were isolated from buffy coats or from umbilical cord blood samples taken immediately following delivery. Adult or cord blood MDM were adherence purified for 7 days in 10% human serum. 24 hours after stimulation with Mtb (iH37Rv; MOI 1-10) or LPS (100 ng/ml) MDM were washed and detached from the plates by cooling and gentle scraping, and placed in flow cytometry tubes. Cells were Fc blocked, exposed to viability dye Zombie NIR and stained with fluorochrome-conjugated antibodies specific for CD14, CD68, CD40, CD80, HLA-DR, CD83, CD86 and MMR (CD206). Cells were analyzed by flow cytometry. The macrophage population was identified on the basis of forward scatter (FSc) and side scatter (SSc). Doublets and dead cells were excluded. Single, live cells were gated on CD68 and CD14 to identify the macrophage population. The mean fluorescent intensity (MFI) of the phenotypic markers for the adult (**A**; n=4 ± SD) and cord (**B**; n=4 ± SD) MDM is shown. Statistical significance was determined using one-way ANOVA with Dunnett’s multiple comparison test; *P < 0.05, **P < 0.01.

In adult MDM, CD40 (a critical costimulatory molecule), HLA-DR (an antigen presenting molecule) and CD83 (activation marker) were all significantly upregulated upon stimulation with Mtb (P<0.05) or LPS (P<0.01) whereas CD80 (costimulatory molecule) was only significantly upregulated in response to LPS (P<0.05) and not in response to Mtb (**Figure 1A**). Changes in CD86 (costimulatory molecule) and MMR (CD206; associated with an “M2” phenotype) expression were not statistically significant (**Figure 1A**). MDM from cord blood significantly upregulated expression of CD40 in response to stimulation with Mtb (P<0.05) and expression of HLA-DR in response to LPS (P<0.01; **Figure 1B**). All other markers were not significantly altered upon stimulation.

These data indicate that adult and cord blood MDM display similar expression of cell surface markers of antigen presentation, maturation and M1/M2 phenotypes.

### The Warburg Effect Occurs Early Post Stimulation in Human Adult but Not Cord Blood Macrophages

**Figure 2:**
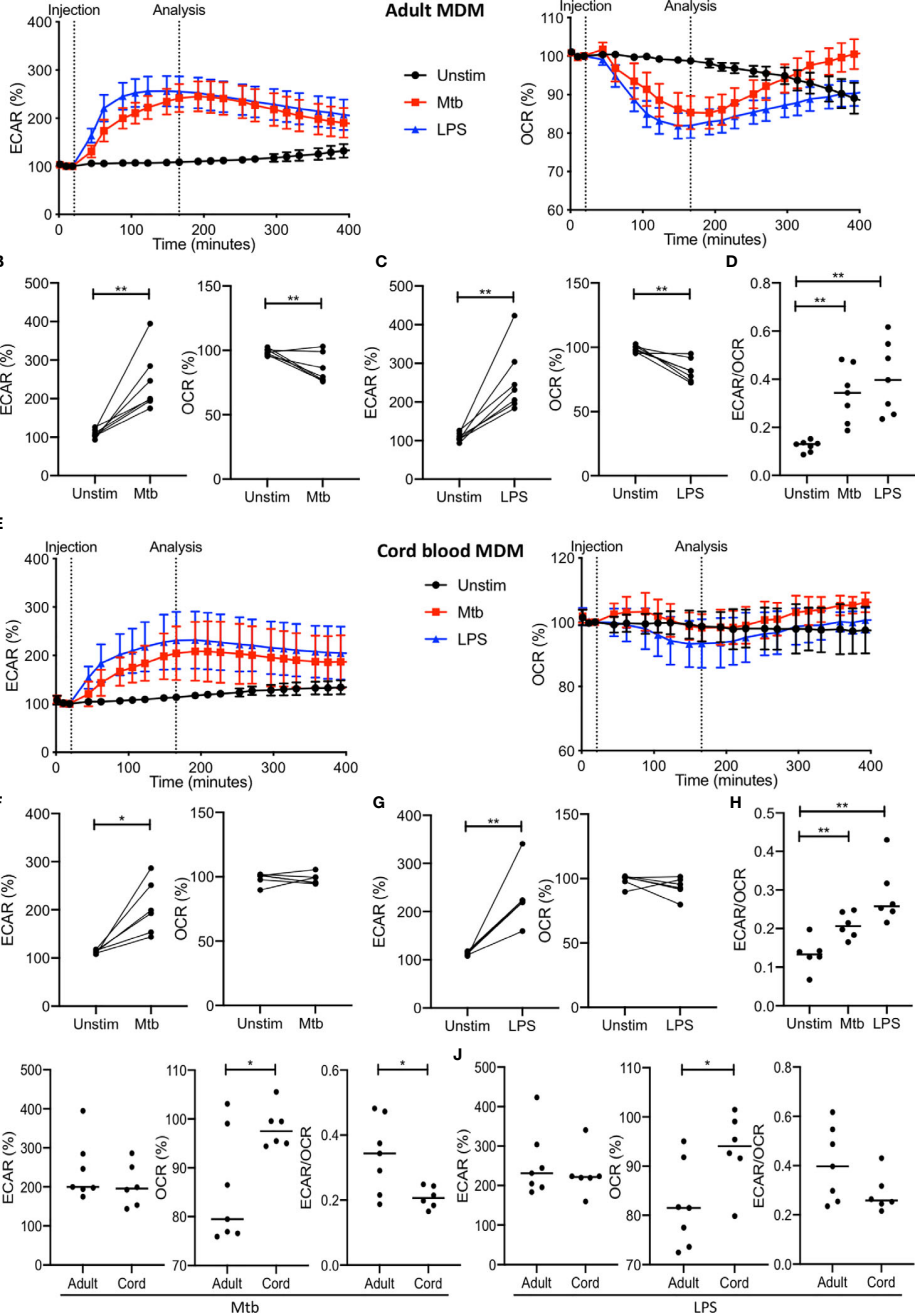
The Warburg effect occurs rapidly in adult MDM after Mtb or LPS stimulation. PBMC were isolated from buffy coats or from umbilical cord blood samples taken immediately following delivery. Adult or cord blood MDM were adherence purified for 7 days in 10% human serum. MDM were washed and detached from the plates by cooling and gentle scraping, counted and re-seeded on Seahorse culture plates prior to analysis in the Seahorse XFe24 Analyzer. Mtb (iH37Rv; MOI 1-10) or LPS (100 ng/ml) were added to the cells in the Seahorse Analyzer. The Extracellular Acidification Rates (ECAR) and Oxygen Consumption Rate (OCR) were graphed approximately every 20 minutes. Correction for variation was achieved by % comparison to the third basal ECAR or OCR reading. After approximately 30 minutes, the Seahorse Analyzer injected medium (unstim), Mtb or LPS into assigned wells. The ECAR and OCR readings were then continually sampled in real time. The time-course graphs illustrate the ECAR and OCR of adult MDM **(A)** or cord blood MDM **(E)** in real time in response to stimulation with Mtb or LPS. At the time point indicated, approximately 150 minutes after stimulation, the ECAR, OCR and the ECAR/OCR ratio were analyzed for Mtb and LPS stimulated cells for the adult (**B–D**; n=7 line at median) and cord blood MDM (**F–H**; n=6 line at median)****. Comparison was made between the adult and cord OCR and ECAR/OCR ratio for the Mtb **(I)** and LPS **(J)** stimulated MDM. Statistically significant differences were determined using a paired **(B, C, F, G)** or unpaired **(I, J)** Student’s t test and one-way ANOVA **(D, H)**, as appropriate; *P < 0.05, **P < 0.01.

In order to examine if the Warburg effect (i.e., increased glycolysis and decreased oxidative phosphorylation) occurs in activated human macrophages, we analyzed the kinetics of the metabolic response of human MDM to stimulation with LPS or Mtb in real time. Since Mtb has been shown to subvert macrophage metabolism (31, 32), we used irradiated H37Rv (iH37Rv) strain of Mtb to elucidate the host response, unperturbed by interference from live, growing Mtb. Using the injection ports of the Seahorse XFe24 Analyzer, we added either LPS or Mtb to wells containing differentiated human adult or cord blood MDM and evaluated the ECAR and the OCR, surrogates for glycolysis and oxidative phosphorylation respectively, approximately every 20 minutes (**Figure 2**). Baseline measurements of ECAR and OCR were recorded for approximately 30 minutes prior to the injection of medium (unstimulated, denoted as “unstim”), Mtb or LPS. The data is expressed as % change from the third baseline reading in order to normalize for differences in human variability and for technical variability in cell seeding density on the Seahorse culture plate.

For the adult MDM, the time course graphs show a rapid increase in ECAR following stimulation with Mtb or LPS compared with the unstimulated control (**Figure 2A**). Concomitantly, a rapid decrease in OCR is observed in adult MDM stimulated with Mtb or LPS but not in the unstimulated control (**Figure 2A**). The elevated ECAR plateaus and persists for the duration of the analyzed time period, however the OCR returns to the baseline rate at approximately 300 minutes. Further analyses were carried out at approximately 150 minutes because the ECAR had consistently plateaued and the OCR was at its lowest point in all donors at this timepoint. Data extracted at the analysis timepoint (indicated on the graph; **Figures 2A, E**), approximately 150 minutes (2.5 hours) post stimulation, from 7 independent experiments, shows that there was a statistically significant increase in ECAR (P<0.01) and a decrease in OCR (P<0.01) in adult MDM stimulated with Mtb (**Figure 2B**) or LPS (**Figure 2C**). In keeping with this, a statistically significant increase in the ECAR/OCR ratio (P<0.01) was observed at this time point for both Mtb and LPS stimulated adult MDM (**Figure 2D**).

In cord blood MDM there is also a rapid increase seen in ECAR following Mtb or LPS stimulation. In contrast to the adult MDM however, there is no change in the OCR (**Figure 2E**). Analysis of the data for n=6 independent cord blood MDM experiments was undertaken at the same time point as for the adult MDM (approximately 2.5 hours post stimulation). There is a significant increase in ECAR in the cord blood MDM stimulated with Mtb (P<0.05; **Figure 2F**) or LPS (P<0.01; **Figure 2G**) but no change in the OCR at this time point in response to either Mtb (**Figure 2F**) or LPS (**Figure 2G**). The increase in ECAR however, still results in a significant change in the ECAR/OCR ratio (P<0.01; **Figure 2H**).

Directly comparing the changes in ECAR or OCR in adult versus cord blood MDM at the indicated time point shows that adult MDM have significantly lower OCR compared with cord blood MDM stimulated with Mtb (**Figure 2I**; P<0.05) or LPS (**Figure 2J**; P<0.05). This is also reflected in the comparative degree of increase of the ECAR/OCR ratio, for cells stimulated with Mtb (P<0.05; **Figure 2I**) or LPS (**Figure 2J**).

These data demonstrate that the Warburg effect occurs in human adult MDM but not in cord blood MDM, and that this change occurs early in response to stimulation and is short lived.

### Human MDM Show Persistent Increases in ECAR 24 Hours After Stimulation

**Figure 3:**
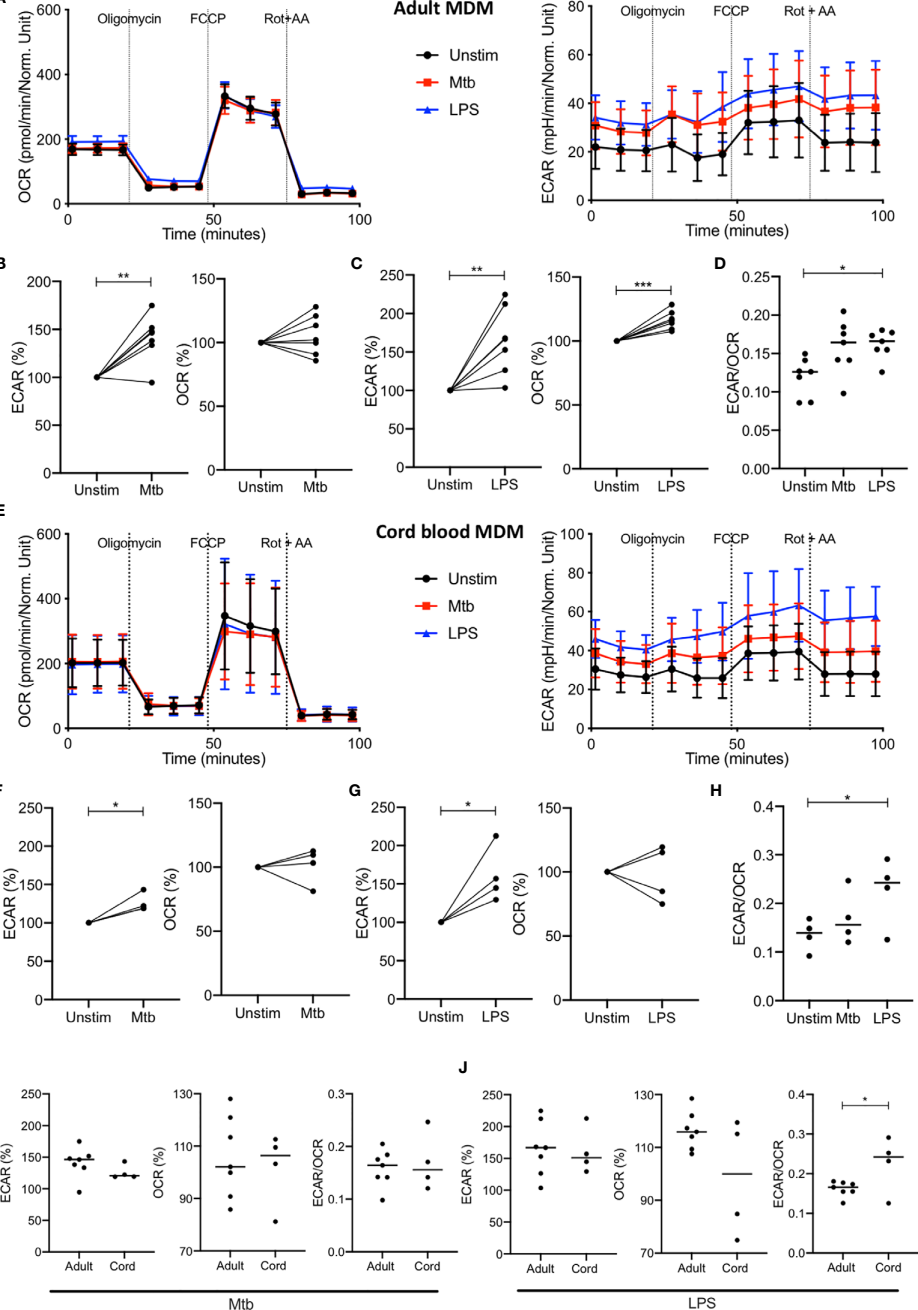
Increase in glycolysis persists at 24 hours but oxidative phosphorylation is no longer decreased in adult MDM. PBMC were isolated from buffy coats or from umbilical cord blood samples taken immediately following delivery. Adult or cord blood MDM were adherence purified for 7 days in 10% human serum. MDM were washed and detached from the plates by cooling and gentle scraping, counted and re-seeded on Seahorse culture plates prior to analysis in the Seahorse XFe24 Analyzer. Mtb (iH37Rv; MOI 1-10) or LPS (100 ng/ml) were added 24 hours prior to mitochondrial stress test analysis for adult **(A)** and cord blood **(E)** MDM. Differences in cell density was corrected for by crystal violet normalization. The extracellular Acidification Rates (ECAR) and Oxygen Consumption Rate (OCR) were recorded approximately every 9 minutes. The baseline ECAR, OCR and the ECAR/OCR ratio were analyzed for Mtb and LPS stimulated cells for the adult (**B–D**; n=7 line at median) and cord blood MDM (**F–H**; n=4 line at median). Comparison was made between the adult and cord OCR and ECAR/OCR ratio for the Mtb **(I)** and LPS **(J)** stimulated MDM. Graphs are expressed as % change from the third baseline reading of unstimulated cells. Statistically significant differences were determined using a paired **(B, C, F, G)** or unpaired **(I, J)** Student’s t test and one-way ANOVA **(D, H)**, as appropriate; *P < 0.05, **P < 0.01, ***P < 0.001.

Previous studies have shown the Warburg effect in murine macrophages at 16-24 hours post activation with LPS but the data at this time point in adult MDM is inconsistent (7, 8, 17). In order to examine the metabolism of adult and cord blood MDM at this later time point, we used the Seahorse XFe24 Analyzer to examine the ECAR and OCR of both adult and cord blood MDM 24 hours after stimulation with Mtb or LPS (**Figure 3**).

The increased ECAR observed 2.5 hours after stimulation with Mtb or LPS persists at 24 hours with basal ECAR for adult MDM remaining increased from baseline (**Figure 3A**, right & B-C; P<0.01). The OCR at 24 hours is distinct for Mtb and LPS stimulation; with adult MDM stimulated with Mtb showing no change in OCR (**Figure 3B**) and those stimulated with LPS showing a significant increase in OCR (**Figure 3C**; P<0.001) compared with unstimulated MDM. The basal ECAR/OCR ratio 24 hours post stimulation is significantly increased in the LPS stimulated cells compared with unstimulated controls (**Figure 3D**; P<0.05). These data indicate that after 24 hours adult MDM stimulated with LPS exhibit enhanced cellular energetics, with both ECAR and OCR elevated. However, MDM stimulated with Mtb for 24 hours exhibit increased ECAR, but stable OCR compared with control, indicating glycolytic metabolism but not a bona fide Warburg effect.

The cord blood MDM 24 hours after stimulation also show a persistent increase in ECAR for both Mtb and LPS (**Figures 3E–G**, P<0.01) with no change in OCR for either Mtb or LPS (**Figures 3E–G**) compared with unstimulated controls. The basal ECAR/OCR ratio is significantly increased in the LPS stimulated cord blood MDM (**Figure 3H**, P<0.05). Direct comparison of the adult and cord blood MDM basal ECAR or OCR shows no difference for either Mtb (**Figure 3I**, left) or LPS (**Figure 3J**, left) stimulation, although the ECAR/OCR ratio for the cord blood MDM is significantly increased compared to the adult MDM 24 hours following LPS stimulation (**Figure 3J**, right; P<0.05).

These data indicate that by 24 hours post infection with Mtb, adult and cord blood MDM exhibit similar cellular energetics. In contrast, when MDM are stimulated with LPS for 24 hours, adult MDM exhibit enhanced cellular energetics whereas cord blood MDM exhibit enhanced glycolysis only. This results in cord blood MDM displaying a significantly increased ECAR/OCR ratio compared with adults, 24 hours post LPS stimulation.

Comparing the ECAR/OCR ratios at 2.5 hours with the ratios at 24 hours post stimulation illustrates that adult MDM have significantly reduced ECAR/OCR ratios at 24 hours post stimulation with Mtb (P<0.001) or LPS (P<0.0001) compared with the earlier timepoint (**Supplementary Figure 2A**). In contrast, cord blood MDM do not have significantly different ECAR/OCR ratios at 2.5 hours compared with those observed at 24 hours post stimulation (**Supplementary Figure 2A**).

Following the basal measurement, a mitochondrial stress test was performed (**Figures 3A, E**). Maximal respiration, spare respiratory capacity, proton leak, ATP respiration and non-mitochondrial oxygen consumption was calculated after the sequential administration of oligomycin (an inhibitor of ATP synthase), FCCP (uncouples mitochondrial oxidative phosphorylation) and rotenone and antimycin A (complex I and complex II inhibitors, respectively: **Figure 3A** and **Supplementary Figure 2B**). Although adult and cord blood MDM responses to the Mito Stress Test were similar, the adult MDM exhibited significant changes in proton leak after both Mtb and LPS stimulation (P<0.05; **Supplementary Figure 2B**) whereas the cord blood MDM did not. As increased proton leak is associated with augmented mitochondrial activity, such as increased oxidative phosphorylation, the inability of Mtb and LPS to increase OCR in cord blood MDM is also reflected by no observed differences in proton leak. Non-mitochondrial oxygen consumption was significantly increased in adult but not cord blood MDM in response to LPS stimulation (P<0.01; **Supplementary Figure 2B**).

These data illustrate the importance of timing when determining immunometabolic responses in human MDM. Although the ECAR remains increased, the early changes in OCR observed at 2.5 hours post stimulation have reversed by 24 hours post LPS stimulation. The fact that these changes are not seen in Mtb stimulated cells highlight the differential metabolic response to different stimuli.

### Cord Blood MDM Produce Less TNF in Response to Mtb and More IL-6 in Response to LPS Stimulation Compared With Adult MDM

Functional impairment of cytokine production in cord blood mononuclear cells (CBMC) is well established (33–35) although there are fewer published data on umbilical cord blood MDM, with contradictory results (22, 24). In addition, cytokine production in macrophages has previously been associated with metabolic function (1, 18, 19, 36). Since neonates are highly susceptible to TB and moreover, have increased risk of disseminated disease and increased mortality, we hypothesized that cord blood and adult MDM may have differential capacity to produce key pro-inflammatory cytokines in response to stimulation with Mtb. Therefore, we examined the concentrations of cytokines including TNF, IL-1β, IL-10 and IL-6, present in the supernatants of MDM from adult or cord blood 24 hours post stimulation with Mtb or LPS.

**Figure 4:**
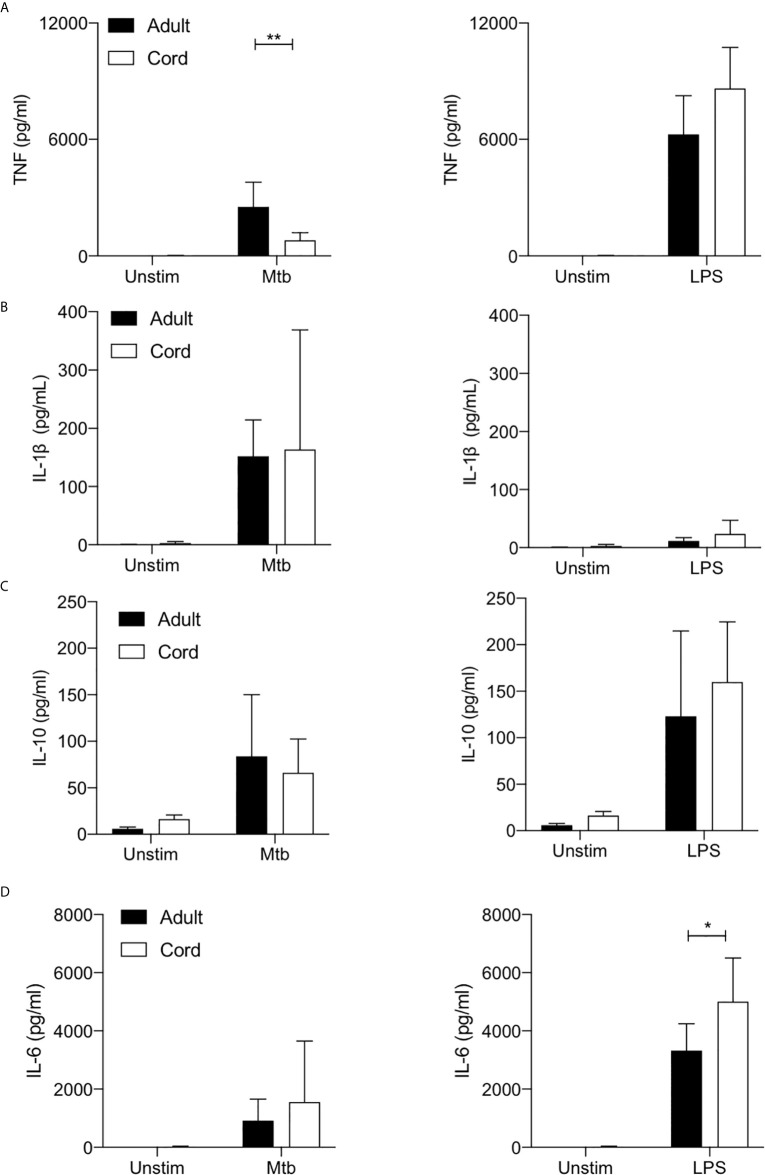
Cord blood macrophages secrete less TNF when stimulated with Mtb stimulation but more IL-6 after LPS stimulation compared with adult macrophages. PBMC were isolated from buffy coats or from umbilical cord blood samples taken immediately following delivery. MDM were adherence purified for 7 days in 10% human serum. The concentrations of TNF **(A)**, IL-1β **(B)**, IL-10 **(C)** and IL-6 **(D)** in supernatants were measured by Mesoscale Discovery assay 24 hours after stimulation with Mtb (iH37Rv; MOI 1-10) or LPS (100 ng/ml). Graphs illustrate collated data from n=4 adult and n=4 cord MDM ± SD. Statistical significance was determined using two-way ANOVA with Sidak’s multiple comparison test; *P < 0.05, **P < 0.01.

TNF is a critical host defense cytokine produced by macrophages in response to stimulation with a wide range of immune stimuli. In the context of TB, TNF plays a crucial role in host defense as evidenced by the reactivation of latent TB in people on anti-TNF therapy (37). Cord blood MDM secreted significantly less TNF than adult MDM in response to stimulation with Mtb (P<0.01; **Figure 4A**). No significant differences were observed between adult and cord blood MDM secretion of TNF in response to LPS stimulation (**Figure 4A**, right).

We have previously reported a critical role for IL-1β in mediating bacterial killing in the context of Mtb infection (17). This production of IL-1β in response to Mtb infection is associated with increased flux through glycolysis (17–19). More broadly, the Warburg effect observed in murine macrophages has been reported to be intrinsically linked to the production of IL-1β (1). Our data indicates that adult and cord blood MDM have similar capacities to secrete IL-1β 24 hours post stimulation with Mtb (**Figure 4B**), although donor-to-donor variability is high in both cord blood and in adults. Unsurprisingly, LPS alone does not significantly induce mature IL-1β in either adult or cord blood MDM (**Figure 4B**, right).

IL-10 is a potent anti-inflammatory cytokine, which promotes immune regulation and is associated with an “M2” phenotype. In the context of TB, induction of IL-10 by Mtb is thought to play a key role in immune evasion (38, 39). Our data shows that both adult and cord blood MDM secrete similar concentrations of IL-10 in response to stimulation with Mtb or LPS (**Figure 4C**).

Finally, IL-6 is a key pyrogenic cytokine with pleiotropic effects including inducing acute phase proteins and the production of neutrophils in the bone marrow. It also plays a pivotal role in regulating Treg/Th17 balance. Our data indicates that adult and cord blood MDM produce comparable concentrations of IL-6 in response to Mtb, but cord blood MDM produce significantly more IL-6 in response to LPS stimulation compared with adult MDM (P<0.05; **Figure 4D**).

In addition, we examined the concentrations of IL-8, IL-12p70 and IFN-γ, however, these cytokines were not statistically significantly induced by stimulation or different in adult compared with cord blood MDM (**Supplementary Figure 3**).

Taken together, these data indicate that cord blood MDM are not generally hyporesponsive in terms of their ability to produce cytokines. Interestingly, cord blood MDM have reduced capacity to produce TNF specifically in response to Mtb but increased ability to produce IL-6 in response to LPS.

## Discussion

The burgeoning field of immunometabolism has great therapeutic potential (18, 19, 40, 41), but specific knowledge gaps exist in basic human macrophage metabolism including definitive evidence that the Warburg effect occurs and whether or not this immunometabolic function is stable over the course of a human lifetime. Translation of immunometabolism into clinical benefit is focused largely on strategies of immunosuppression. We propose that better understanding of human immunometabolism may also provide druggable targets for bolstering immune responses - in contexts where immuno-supportive therapy is required. The previously identified discrepancies in murine and human models highlight the need for caution in drawing conclusions on therapeutic efficacy from animal studies (7, 8). Our hypothesis driven human research aimed to close these two key knowledge gaps by examining the immunometabolic response of human macrophages stimulated in real time and by comparing the metabolic responses of adult with cord blood MDM.

We show for the first time that the Warburg effect occurs in human adult primary macrophages within the first hours after stimulation and then resolves. Twenty-four hours after stimulation, the cells maintain an increase in glycolysis but the reduction in oxygen consumption has returned to baseline in Mtb-stimulated macrophages and is unexpectedly increased after LPS stimulation. We also demonstrate, for the first time, that cord blood MDM do not shift to Warburg metabolism early in the response to stimulation but display similar energetic profiles compared with adults by 24 hours post stimulation.

Macrophage effector function is vital for Mtb control (42). Neonates are 5-10 fold more likely to develop infection (43, 44) following exposure and alveolar macrophages from newborns are unable to control Mtb (45). We hypothesized that immunometabolic differences between adult and neonates exist and that a metabolic impairment in neonates may play a role in susceptibility to infection. We have shown that in contrast to adult MDM, MDM from cord blood fail to reduce oxidative phosphorylation upon stimulation.

Deficiencies in immune cells from umbilical cord blood have been attributed to the altered immune environment required for fetal growth, in particular high levels of migratory inhibitory factor and adenosine in cord blood serum (46). In our system, the adult and cord blood MDM have been differentiated in the same manner, in the presence of pooled adult human serum. This process also requires multiple washings of non-adherent cells and medium changes which results in experiments being performed free from any persisting environmental factors. This method also does not polarize the cells, which may explain the differences between our data and another study which showed that IFN-γ or IL-10 polarized cord blood MDM failed to increase glycolysis during a glycolysis stress test (25). We report that cord blood MDM can effectively increase glycolysis. Since the ability to shift to glycolysis is intrinsically linked with IL-1β secretion (17), the finding that cord blood MDM can undergo a shift to glycolysis is consistent with our data, and with previously published data (47, 48), showing that cord blood and adult MDM make similar amounts of IL-1β. However, we cannot conclude from our data that these two phenomena are directly linked.

Previous studies on cytokine production in CBMC report a decrease in TNF production compared with adult PBMC (49, 50). One study examining cord blood monocytes using LPS and ﻿lipoarabinomannan, a component of the mycobacterial cell well, showed a decrease in TNF production 6 hours after stimulation (51). However, there is no previously published data to our knowledge examining the ability of cord blood MDM to produce TNF in response to stimulation with Mtb. We observed significantly reduced TNF in cord blood MDM stimulated with Mtb compared with adults; this novel finding may, at least in part, account for increased susceptibility to Mtb infection in neonates and warrants further exploration in clinical settings.

Production of TNF is not thought to be metabolically mediated, as evidence from mice suggests that TNF remains stable when glycolysis (and therefore IL-1β production) is being modulated (1). Whilst this phenomenon may be distinct and unrelated from the altered metabolic profile, our previously published data (18, 19) suggested that TNF may be linked to metabolic function in humans and therefore warrants further study. Timing and the kinetics of metabolic flux versus cytokine production may be imperative to understanding how these processes are connected in humans. Since our data examined the concentrations of cytokines present and accumulated over a 24-hour period, we cannot rule out that differences may exist in the early production of cytokines or at the level of gene expression.

Interestingly, the data indicates that cord blood MDM have enhanced ability to produce IL-6 in response to LPS compared with adults. This is in keeping with published work showing increased IL-6/TNF ratios in cord blood versus adult PBMC (50).

We also observed similar morphology and phagocytic ability in cord and adult MDM, as previously described (22, 52, 53). Cord blood MDM were more prone to death following exposure to cold and then gentle scraping. This was adjusted for in our experiments to ensure numbers were equal, however, it is plausible that cord blood MDM may exhibit increased susceptibility to death following stress compared with adult MDM.

Our flow cytometry based phenotypic analysis of adult and cord blood MDM showing similar expression of cell surface markers, supports the results of previous studies indicating that monocytes or macrophages from cord blood are phenotypically similar to adults and are able to respond appropriately to stimulation in certain contexts (23, 24, 33). At this timepoint, adult and cord blood MDM metabolic function are very similar and since metabolic function is associated with ‘M1/M2’ phenotypes, it is therefore unsurprising that ‘M1/M2’ markers are broadly similar in adult and cord blood MDM. Although cord blood MDM stimulated with LPS are more energetic at 24 hours than their adult counterparts, this is not reflected in differences in their cell surface expression of key activation markers.

### Study Limitations

Although we have shown that human macrophages undergo early Warburg metabolism, the importance of this mechanism in a human system is yet to be fully understood. Inhibition of glycolysis is known to inhibit IL-1β production, but the downstream effect on macrophage activation and function of a decrease in oxidative phosphorylation is beyond the scope of this current study. It is hypothesized in murine models that the break in the tri-carboxylic acid cycle is required in order to increase immunomodulatory substrates such as succinate and itaconate. Whether the differences in oxygen consumption seen in adult and cord blood MDM relates directly to the altered immunophenotype will be an area of future study. Evidence in the literature suggests that metabolic function is directly linked to cytokine production, however, we acknowledge that our data does not mechanistically link observations in metabolic function with cytokine output.

In order to fully explore the impact of immunometabolism in the context of Mtb, infection with live, replicating mycobacteria will be required. Our model focuses on the host response to infection, which live Mtb has been shown to subvert. For this reason, we used irradiated H37Rv to assess the metabolic response of the host independent of bacterial evasion strategies. However, since different strains of live Mtb have been shown to differentially manipulate immunometabolic responses (54), future translational research will need to take this into account. We also recognize that while neonatal immune responses to Mtb may be insufficient and therefore result in disseminated disease with increased severity and mortality, strategies to boost immune responses may be detrimental in certain contexts of active TB disease where pathology may be immune mediated.

Neonatal and pediatric research is limited by small blood donation volumes, so using umbilical cord blood overcomes this issue. However, a knowledge gaps exist around how closely cord blood recapitulates neonatal immune responses in early life. Furthermore, the mode of delivery can impact the immune responsiveness of cord blood (55). By using umbilical cord blood from planned Caesarean sections only and by differentiating the monocytes *in vitro* with the same adult human serum used to differentiate adult MDM for 7 days, we aimed to overcome some of the confounding unknowns in this model and increase the comparability of MDM from adult and cord blood.

In order to translate these findings into effective immuno-supportive medicine, tailored specifically to populations vulnerable to infection at the poles of the human lifespan, further studies are ongoing to elucidate immunometabolic alterations in ageing adults.

## Conclusion

Adult human macrophages exhibit the Warburg effect rapidly post stimulation and maintain an elevated rate of glycolysis (but not the decreased oxidative phosphorylation) 24 hours later. This is in contrast to the metabolic flux described in murine models, which exhibits Warburg metabolism 24 hours post stimulation with LPS. The data presented in the current study highlights the importance of assessing immunometabolic kinetics in primary human immune cells. We have shown that there is a fundamental metabolic difference in MDM from umbilical cord blood and adult blood, despite using the same method of differentiation from monocytes. Furthermore, we report that cord blood MDM produce significantly less TNF in response to Mtb compared with adult MDM. These key differences in early metabolic flux and cytokine production may help to determine why certain populations are more vulnerable to infection than others and may lead to the development of specific immuno-supportive therapies for susceptible people.

## Data Availability Statement

The raw data supporting the conclusions of this article will be made available by the authors, without undue reservation.

## Ethics Statement

The studies involving human participants were reviewed and approved by The Rotunda Hospital Research and Ethics Committee and by Trinity College Dublin School of Medicine Research Ethics Committee. Written informed consent to participate in this study was provided by the participants’ legal guardian/next of kin.

## Author Contributions

CÓM: conceptualization, methodology, formal analysis, investigation, writing - original draft, visualization, project administration and funding acquisition. DC: methodology, formal analysis, investigation, visualization, writing - review and editing. JP: investigation, writing - review and editing. FM: resources, writing - review and editing. JK: conceptualization, writing - review and editing, supervision and funding acquisition. SB: conceptualization, formal analysis, investigation, writing - original draft, writing - review and editing, visualization, supervision and funding acquisition. All authors contributed to the article and approved the submitted version.

## Funding

This work was funded by The National Children’s Research Centre (D/18/1) and by The Royal City of Dublin Hospital Trust.

## Conflict of Interest

The authors declare that the research was conducted in the absence of any commercial or financial relationships that could be construed as a potential conflict of interest.
