# Supplementary material for: The Warburg Effect Occurs Rapidly in Stimulated Human Adult but Not Umbilical Cord Blood Derived Macrophages
Source: Front Immunol. 2021 Apr 13;12:657261. doi: 10.3389/fimmu.2021.657261 (PMC8076563; doi:10.3389/fimmu.2021.657261)
Supplement: Supplementary file 1 [file DataSheet_1.pdf]

Supplementary Figures

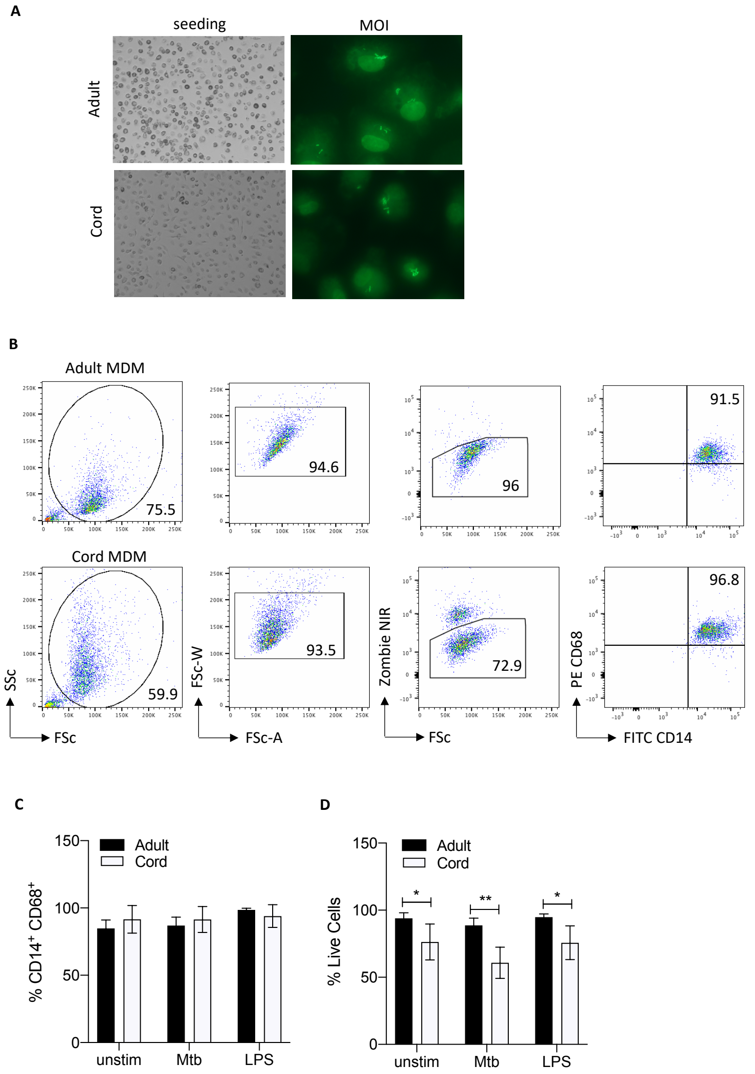

Supplementary Figure 1. Adult and cord blood MDM morphology, phagocytosis, viability and purity following differentiation. PBMC were isolated from buffy coats or from umbilical cord blood samples taken immediately following delivery. Adult or cord blood MDM were adherence purified for 7 days in 10% human serum. Light microscopy images of adult and cord (A) MDM were taken. Prior to stimulation with Mtb a multiplicity of infection was performed for each donor by adding variable amounts of Mtb to MDM on 8 well labteks. After 3 hours the MDM were washed and stained with Hoechst and auramine o dye. Using fluorescent microscopy, the percentage of cells infected and the numbers of bacilli per cell were recorded and used to calculate the volume added to the experimental wells (A). MDM were washed with cold PBS, cooled on ice for 30 minutes and cells were then gently scraped and placed in flow cytometry tubes. MDM were exposed to Zombie NIR viability dye, Fc blocked and stained with fluorochrome-conjugated antibodies specific for CD14 (FITC) and CD68 (PE) and acquired by flow cytometry. The dot plots show the gating strategy (B). The graphs illustrate collated data for n=4 cord blood and n= 4 adult MDM; purity of the MDM population on day 7 of differentiation (C) and viability after cold lifting and scraping (D). Statistical significance was determined using two-way ANOVA with Sidak's multiple comparison test; * $P<0.05$, ** $P<0.01$.

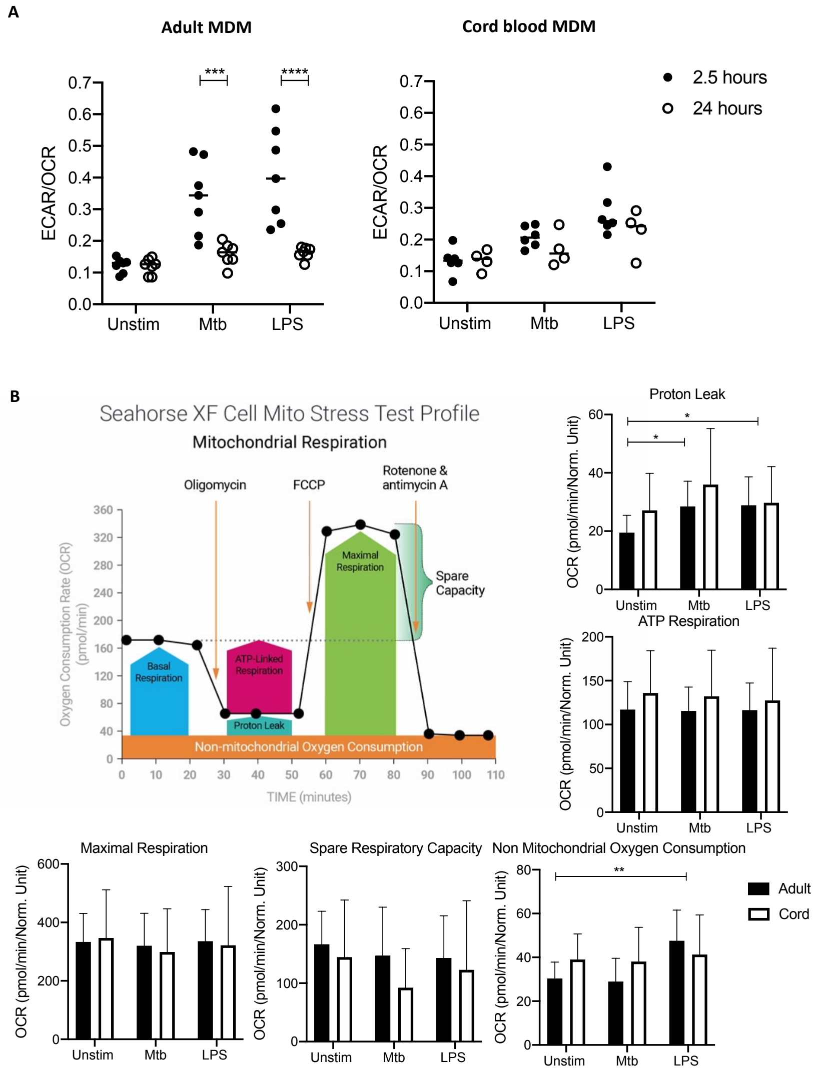

Supplementary Figure 2. ECAR/OCR ratios at different time-points and the mitochondrial stress test data. PBMC were isolated from buffy coats or from umbilical cord blood samples taken immediately following delivery. Adult or cord blood MDM were adherence purified for 7 days in 10% human serum. MDM were washed with cold PBS, cooled on ice for 30 minutes and then gently scraped, counted and re-seeded on Seahorse culture plates prior to analysis in the Seahorse XFe24 Analyzer. Mtb (iH37Rv; MOI 1-10) or LPS (100 ng/ml) were added in real time in the Seahorse XFe24 Analyzer or 24 hours prior to mitochondrial stress test analysis for adult and cord blood MDM. (A) Graphs show the differences in the ECAR/OCR ratios at 2.5 hours versus 24 hours post stimulation (n=4-7). (B) The Mitochondrial Stress test profile from the manufacturer (Agilent) is shown in the schematic. Drugs that modulate mitochondrial function were introduced to the cells as shown. Differences in cell densities were corrected for by crystal violet normalization. The extracellular Oxygen Consumption Rate (OCR) was recorded approximately every 9 minutes. The mitochondrial stress test allows the calculation of the Spare Respiratory Capacity, ATP respiration, Proton Leak, Maximal respiration and Non-Mitochondrial Oxygen Consumption which are illustrated in the graphs for both adult and cord blood MDM (n=4 for both adult and cord ±SD). Statistical significance was determined using two-way ANOVA with Sidak's multiple comparison test * $P<0.05$, ** $P<0.01$, *** $P<0.001$, **** $P<0.0001$.

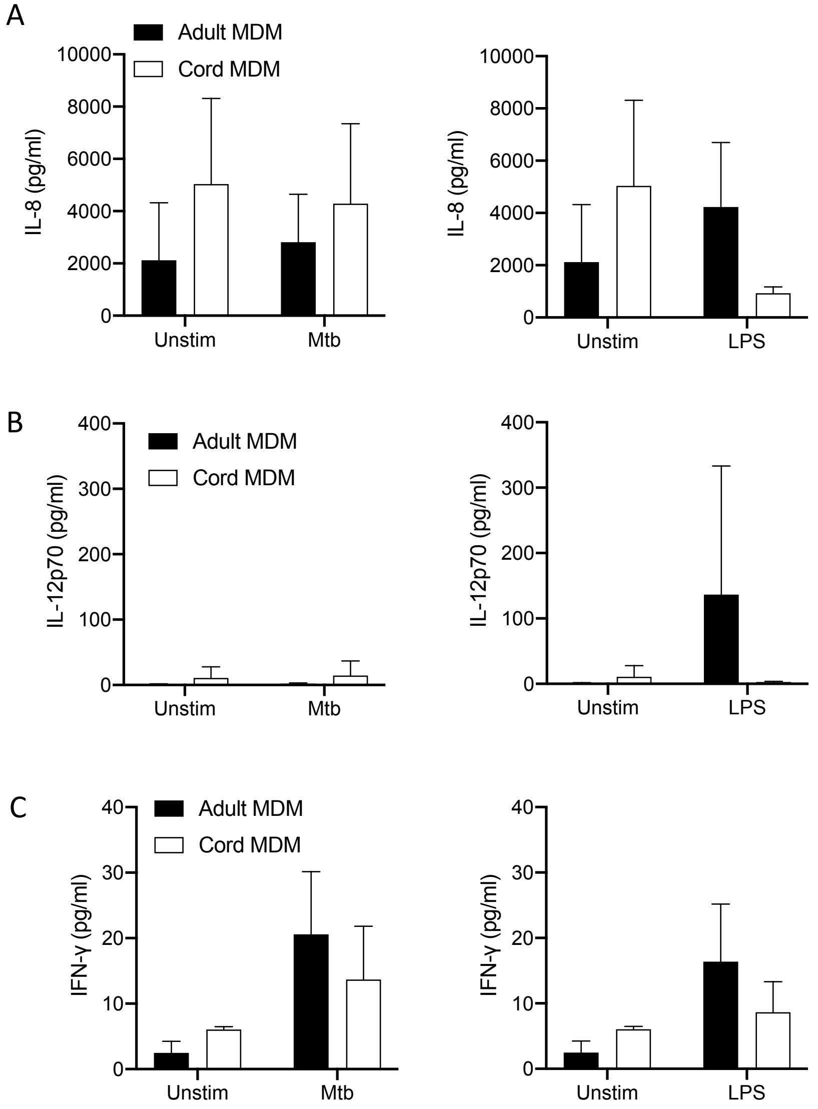

Supplementary Figure 3. Cytokine production from adult and cord blood MDM. PBMC were isolated from buffy coats or from umbilical cord blood samples taken immediately following delivery. MDM were adherence purified for 7 days in 10% human serum. The concentrations of IL-8 (A), IL-12p70 (B) and IFN-γ (C) in supernatants were measured by Mesoscale Discovery assay 24 hours after stimulation with Mtb (iH37Rv; MOI 1-10) or LPS (100 ng/ml). Graphs illustrate collated data from n=4 adult and n=4 cord MDM ±SD. Statistical significance was determined using two-way ANOVA with Sidak's multiple comparison test; results were not statically significant.
